# Cerebrovascular Blood Flow Design and Regulation; Vulnerability in Aging Brain

**DOI:** 10.3389/fphys.2020.584891

**Published:** 2020-10-16

**Authors:** David F. Wilson, Franz M. Matschinsky

**Affiliations:** Department of Biochemistry and Biophysics, Perelman School of Medicine, University of Pennsylvania, Philadelphia, PA, United States

**Keywords:** neural activation, BOLD effect, brain oxygenation, cortical vasculature, aging brain

## Abstract

Nutrient delivery to the brain presents a unique challenge because the tissue functions as a computer system with in the order of 200,000 neurons/mm^3^. Penetrating arterioles bud from surface arteries of the brain and penetrate downward through the cortex. Capillary networks spread from penetrating arterioles through the surrounding tissue. Each penetrating arteriole forms a vascular unit, with little sharing of flow among vascular units (collateral flow). Unlike cells in other tissues, neurons have to be operationally isolated, interacting with other neurons through specific electrical connections. Neuronal activation typically involves only a few of the cells within a vascular unit, but the local increase in nutrient consumption is substantial. The metabolic response to activation is transmitted to the feeding arteriole through the endothelium of neighboring capillaries and alters calcium permeability of smooth muscle in the wall resulting in modulation of flow through the entire vascular unit. Many age and trauma related brain pathologies can be traced to vascular malfunction. This includes: 1. Physical damage such as in traumatic injury with imposed shear stress as soft tissue moves relative to the skull. Lack of collateral flow among vascular units results in death of the cells in that vascular unit and loss of brain tissue. 2. Age dependent changes lead to progressive increase in vascular resistance and decrease in tissue levels of oxygen and glucose. Chronic hypoxia/hypoglycemia compromises tissue energy metabolism and related regulatory processes. This alters stem cell proliferation and differentiation, undermines vascular integrity, and suppresses critical repair mechanisms such as oligodendrocyte generation and maturation. Reduced structural integrity results in local regions of acute hypoxia and microbleeds, while failure of oligodendrocytes to fully mature leads to poor axonal myelination and defective neuronal function. Understanding and treating age related pathologies, particularly in brain, requires better knowledge of why and how vasculature changes with age. That knowledge will, hopefully, make possible drugs/methods for protecting vascular function, substantially alleviating the negative health and cognitive deficits associated with growing old.

## An Overview: Brain Function and Vascular Design

Regulation of nutrient delivery to the brain presents a unique challenge because, unlike cells in other tissues, individual neurons must be isolated from each other except as they interact through electrical connections. Interactions other than through the electrical connections reduce the fidelity of neural function, i.e., reduce the accuracy with which the efferent neural activity is an appropriate response to input. In other tissues, such as muscle and liver, cells work together to carry out a common or closely related functions and there is extensive cell-cell interaction. In brain, however, each neuron has to operate independent of neighboring neurons except through electrical connections. This difference is important to understanding the metabolic and vascular responses in brain and how they are similar to, and different from, those in other tissues. As will be developed in this paper, the functional characteristics of brain give rise to an apparent mismatch between the vascular system and metabolic requirements. The smallest vessels with regulated blood flow are arterioles that feed the capillaries. In brain, blood flow through each arteriole provides nutrients to, and removes waste products from, a volume of tissue that contains 7,000 to 20,000 individual neurons. Neural stimulation, such as somatosensory stimulation, activates only a fraction of the neurons fed by an individual arteriole and these are not uniformly spread through the tissue. As a result, activation gives rise to inhomogeneity in metabolic requirements, with relatively large changes occurring in tissue volumes that are much smaller than the volume fed by an arteriole. Blood flow must increase sufficiently to meet the requirements of the activated neurons, but increase in flow through an arteriole is distributed to all of the tissue fed by the arteriole. Increase in flow sufficient to meet the increase in oxygen of the activated neurons also increases delivery to the tissue regions where the neurons are not activated. The result is an apparent global flow-consumption mismatch in which increased oxygen delivery appears to substantially exceed the increase in oxygen consumption.

Nutrients are delivered, and byproducts removed, by blood pumped through the body by the heart, which accepts deoxygenated blood from the veins, sends it to the lungs for reoxygenation and removal of CO_2_, and then sends refreshed blood to tissues through the arterial system. The normal output pressure from each heart beat is from 60 to 120 mm Hg. Arteries have walls consisting of multiple layers of cells, endothelial cells covered by smooth muscle. Flow is progressively divided among increasing numbers of smaller and smaller vessels with thinner and thinned walls, forming a highly branched distribution system. The walls provide the strength and elasticity necessary to contain pressurized liquid as well as smooth muscle for regulating vessel diameter (flow resistance). Flow is distributed among the branches as needed for delivery of nutrients to the tissue fed by that particular vessel through dynamic modulation of vessel diameters. Rhodin (1967) proposed “*the term arteriole be used for small arteries ranging approximately between 100 μ and 50 μ. Thus, an arteriole would have more than one smooth muscle layer, a well developed elastica interna, and nerves which are associated mainly with the outermost smooth muscle layer. A terminal arteriole is a vessel with a diameter of less than 50 μ which has only a single muscle layer, frequent membranous contacts between endothelium and muscle cells (myoendothelial junctions), a scanty or mostly absent elastica interna, and nerves accompanying it very closely. The term precapillary sphincter should be reserved for the muscular arrangements around the orifices of the smaller branches which come off the terminal arterioles. The diameter of the precapillary sphincter may vary from 7 μ to about 15 μ, but it is characterized by an arrangement of smooth muscle cells which have particularly numerous, often club-shaped contacts with the endothelium (myoendothelial junctions).*” This general description and nomenclature are appropriate for cerebral vasculature but it should be noted that cerebral vasculature does not have precapillary sphincters. Contemporary descriptions of the vasculature can be found in a volume of Current Topics in Membranes edited by Jackson (2020).

Capillaries, in contrast to arterioles, have walls consisting of a single layer of endothelial cells. The thin walls of capillaries have limited ability to contain liquid when internal pressures exceed that in the interstitial space. In human capillaries, the osmotic pressure of blood plasma is about 28 mm Hg, which tends to keep fluid in the capillaries. The forces acting on the fluid include hydrostatic pressure of about 20 mm Hg, interstitial-fluid osmotic pressure of about 8 mm Hg, and interstitial fluid hydrostatic pressure, which is normally −1 to −3 mm Hg (Guyton et al., 1971; Aukland and Reed, 1993). These pressures keep the capillaries open and the outside negative pressure results in a small net fluid flow through capillary walls into surrounding tissues (lymph flow). To reach the tissue, nutrients have to cross the vessel wall, oxygen by diffusion and others, such as glucose, through specific transporters. The oxygen concentration gradient across the wall has been measured for vessels of different sizes and found to be similar to that in the surrounding tissue (Vovenko, 1999; Sharan et al., 2008). As a result, mean metabolic activity of the tissue is directly related to vascular surface area. The surface area of capillaries greatly exceeds that of the rest of the vascular system, providing 65–80% of total surface area (Lauwers et al., 2008). The combination of large surface area, high gas permeability, and extensive transporters for metabolites, gives capillaries primary responsible for delivering nutrients to, and collecting waste products from, tissue metabolism. Capillaries empty into collecting veins and blood returns to the heart through progressively larger veins.

Blinder et al. (2010) determined the topological distribution of vessels fed by the middle cerebral artery. The surface arteries form a network with interconnected loops. Interconnections in the loops distribute flow among the vessels such that occlusion of an individual artery slows, but does not stop, local flow. This prevents occlusion of an individual artery within the network from resulting in severe hypoxia in a relatively large region of the brain. Nutrients are provided to the tissue through penetrating arterioles which bud from the surface arteries and travel nearly straight down into and through the cortex. The penetrating arterioles are less than 50 μm in diameter. The smaller and shorter vessels feed the surface layer of the cortex while the larger and longer pass through the cortex to also feed the white matter. Each penetrating arteriole, its capillaries, and collecting veins functions as a distinct vascular unit. Capillaries bud from each penetrating arteriole and spread outward through the surrounding tissue. Cortical tissue is organized in layers, each with different but highly correlated neuronal density, content of cytochrome c oxidase, metabolic activity, and blood flow (Duvernoy et al., 1981; Cox et al., 1993; Lauwers et al., 2008; Adams et al., 2015).

Capillary density also differs from layer to layer and region to region, the number per unit volume closely related to local metabolic activity. Although capillaries radiating from different penetrating arterioles overlap at the edge of their respective tissue columns, there is little or no “mixing” of flow between penetrating arterioles (Cox et al., 1993; Lauwers et al., 2008; Blinder et al., 2010, 2013; Nishimura et al., 2010; Adams et al., 2015). Occlusion of a penetrating arteriole, in contrast to occlusion of a vessel in the network of surface arteries, results in death of cells throughout the column of tissue fed by that arteriole. Blinder et al. (2010, 2013) determined the extent of cell death and cyst formation following coagulation of individual penetrating arterioles and concluded that each arteriole feeds a tissue volume of approximately 0.17 mm^3^ (Blinder et al., 2010, 2013). This is in good agreement with the observation by Silver (1978) that the increase in tissue oxygen pressure following local stimulation is limited to a region within about 250 microns of the stimulation site.

### Oxygen Distribution in the Cerebral Vasculature

The cardio-pulmonary-vascular system is designed to provide tissue with nutrients, and extensive efforts have been devoted to measuring the levels of critical nutrients in tissues. Oxygen is the nutrient required in greatest amounts and for which there is minimal storage capacity, so vascular design and regulation are optimized for oxygen delivery. This delivery has a non-vascular component, oxygen diffusion from the vascular lumen to the mitochondria where it is consumed, and pO_2_ needs to be sustained at levels suitable for cellular function and survival. As blood passes through the arterial tree and capillaries, pO_2_ and oxygen content decrease. The extent of decrease as well as the concentration gradients formed have been largely established for control conditions. Vovenko (1999) provided a detailed account of intravascular oxygen pressure in cerebral vessels of rats. As arterial branching increased from 1^*o*^ to 5^*o*^, vessel diameter decreased from 46 to 8 μm. The pO_2_ and oxygen saturation of hemoglobin in the red cells decreased with increased branching (decreasing vessel diameter), with pO_2_ falling from 81 torr to 62 torr and hemoglobin oxygen saturation from 94 to 84%. Most of the precapillary decrease in hemoglobin saturation (11%) occurred in the 5^*o*^ branch vessels (8 μm diameter). By the venous end of capillaries, pO_2_ had decreased to a mean value of 38 torr and hemoglobin saturation to 54%. Measurements of pO_2_ along capillaries in the direction of flow show decrease in the form of a truncated cone around the vessel (Vinogradov and Wilson, 1994; Senatorov et al., 2019) as proposed by Krogh (1919). The pO_2_ in blood exiting capillaries and entering veins is quite variable but a mean near mixed venous value. There is no significant delivery of oxygen to tissue from veins, indeed pO_2_ in venous blood may actually increase slightly as veins pass near arterioles or through tissue where pO_2_ is higher than in venous blood. Overall, about 45% of the oxygen in blood when it leaves the heart is delivered to tissue.

### Oxygen Distribution in Brain Tissue

Concentration gradients form wherever there is a separation in space between source and sink for a nutrient. For oxygen, this is the distance between capillary blood plasma and mitochondria. Mitochondria are in cells and effectively randomly distributed throughout tissue. As noted earlier, in the hands of skilled researchers, electrodes have been quite successful in measuring pO_2_ in soft tissues. It is an invasive method, however, and insertion causes damage along the path of insertion and applied pressure can suppress blood flow. Both tissue damage and compression errors have been minimized by using ultra small tipped (<5 micron) high resolution electrodes (Silver, 1965; Vovenko, 1999) and special insertion techniques (Baumgärtl et al., 2002), but the method is still limited in application. Development of an accurate and non-invasive optical method for measuring oxygen in tissue *in vivo*, oxygen dependent quenching of phosphorescence (Vanderkooi et al., 1987; Wilson et al., 1988; Pastuszko et al., 1993; Vinogradov and Wilson, 1995; Dunphy et al., 2002; Finikova et al., 2008; Sakadžić et al., 2010; Esipova et al., 2011, 2019; Devor et al., 2014; Esipova and Vinogradov, 2014), is greatly extending our understanding of tissue oxygenation and oxygen delivery to tissue. This method is based on oxygen dependent quenching of the phosphorescence of specially designed molecules (Oxyphors) and is highly specific for oxygen, the only quenching agent with significant concentrations in tissues. When an Oxyphor is excited by light absorption, energy from the absorbed photon raises the porphyrin to an excited triplet state and it returns to the ground state by emitting light (phosphorescence or delayed fluorescence). While in the excited state, collision with an oxygen molecule can result in transfer of energy from the triplet state to oxygen, quenching light emission. The quenching follows well defined physical principles as described by the Stern-Volmer equation:

(1) $${\text{t}}^{\text{o}}/\text{t}=1+{\text{K}}_{\text{Q}}\times t^{\text{o}}\times {\mathrm{pO}}_{2}$$

where t and t^o^ are the phosphorescence lifetime and the lifetime at zero oxygen, respectively. K_Q_ is the quenching constant and pO_2_ the oxygen pressure. Measurements of phosphorescence lifetime are not sensitive to the presence of other chromophors or fluorophors, which can lower the signal to noise ratio (SNR) but do not introduce systematic errors in measurements of phosphorescence lifetime. Intrinsic distributions of phosphorescence lifetimes of phosphorescent probes are narrow compared to the distribution of lifetimes observed in normoxic tissues. As a result, the distribution of lifetimes of oxygen sensitive phosphors in tissues can provide a measure of the distribution of oxygen in that tissue (Vinogradov and Wilson, 1994; Vinogradov et al., 2001; Schears et al., 2005; Wilson et al., 2006). Measurements of Oxyphor in the interstitial space show pO_2_ has a skewed Gaussian distribution peaking at 30–40 torr with little or no significant tissue volume below 10–15 torr (Schears et al., 2005; Wilson et al., 2006; Sakadžić et al., 2010). This is in good agreement with distributions measured with oxygen electrodes (Baumgärtl et al., 2002; Sakadžić et al., 2010; Senatorov et al., 2019). Histograms of pO_2_ in the microvasculature and interstitial space in muscle of anesthetized rats, measured by phosphorescence quenching, show minimal differences in the lower pO_2_ region (Wilson et al., 2006). The lowest pO_2_ regions of the histograms reflect minimal pO_2_ values in capillaries (vascular blood plasma) and in the interstitial space. The latter must be lower than the former with the difference a measure of the diffusion gradient between capillary blood plasma and pericellular surface. In resting muscle, this is <2 torr, indicating the diffusion gradient between capillary blood plasma (source) and pericellular space (sink) is less than about 2 torr. Vovenko (1999) and Sharan et al. (2008) used fine tipped oxygen electrodes to measure the difference in pO_2_ across the walls of rat cortical arterioles and in tissue at progressively greater distances from the vessel wall. Sharan et al. (2008) concluded that pO_2_ gradients across vessel walls were not different from those in the surrounding tissue; i.e., the rates of oxygen consumption and oxygen diffusivity in the wall are not substantially different from those in the interstitial space. The measured gradient as oxygen diffused away from the vessel into the tissue (∼1 torr/μm) is greater than that in resting muscle, consistent with the higher metabolic activity in brain. It is important to note that the measurements were made are for arterioles with pO_2_ much above that in the surrounding tissue. This does not mean pericellular pO_2_ for cells 12 μm from a capillary is 12 torr less than that in the neighboring capillaries. These cells receive oxygen from 3 to 4 different capillaries and the gradient at that point is near zero. The pO_2_ difference between the pericellular space and mitochondria has been measured indirectly in isolated neuroblastoma cells (Robiolio et al., 1989) and directly in lymphocytes (Finikova et al., 2008) and is small, <2 torr, in resting cells.

## Vascular Design and Regulation for Support of Brain Function

**FIGURE 1:**
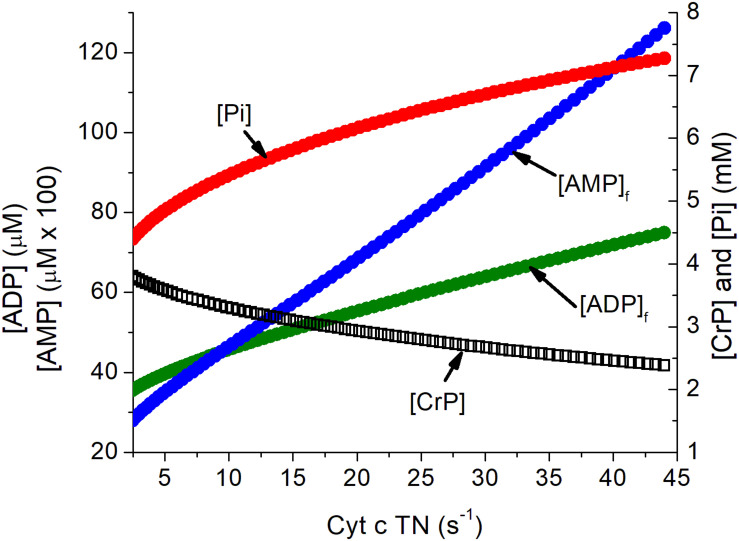
Simulation of the relationship of the concentration of energy metabolites to cytochrome c turnover (oxygen consumption). Simulation was carried out as described previously (Wilson, 2017). The concentrations of ATP, total creatine (CrP + Cr), and cytochrome c ([cyt c]), were assumed to be 4.5 mM, 8 mM, and 8 μM, consistent with experimental measurements in brain tissue. Values for [AMP]_f_ and [ADP]_f_ assumed equilibrium for adenylate kinase (Keq = 1.0) and creatine kinase (Keq = 140). The cytochrome c turnover number can be converted to oxygen consumption by multiplying by [cyt c] and dividing by 4, i.e., a turnover number of 10 s^–1^ corresponds to oxygen consumption of 20 μM s^–1^, 1.2 mM/min, or 2.7 ml/100 g s^–1^. The assumed [cyt c] of 8 μM is conservative and could be as high as 12 μM.

Activation of a neuron imposes a substantial metabolic load on its cells, increasing the rate of ATP consumption by several fold both in the cell bodies and the synapses of its connected dendrites (Ashrafi and Ryan, 2017; Ashrafi et al., 2017). The metabolic changes in ATP, ADP, Pi, and AMP in neurons in response to increase in ATP consumption has been simulated as previously described (Wilson et al., 1979; Wilson and Matchinsky, 2019) and are presented in Figure 1. The assumed increase when neurons are activated exceeds the mean increase in the tissue oxygen consumption observed with somatosensory stimulation but is consistent with this activation involving a limited fraction of the neurons in the tissue. The concentrations of creatine phosphate ([CrP], creatine ([Cr]), and inorganic phosphate ([Pi]) are consistent with published measurements while those of free ADP ([ADP]_f_) and adenosine monophosphate ([AMP]_f_) were calculated assuming equilibrium for creatine kinase and adenylate kinase. The metabolite changes are substantial and regulatory responses include AMP dependent protein kinase (AMPK) dependent increase in GLUT4 incorporation into the plasma membrane (Ashrafi and Ryan, 2017; Ashrafi et al., 2017; Wilson and Matschinsky, 2019).

**FIGURE 2:**
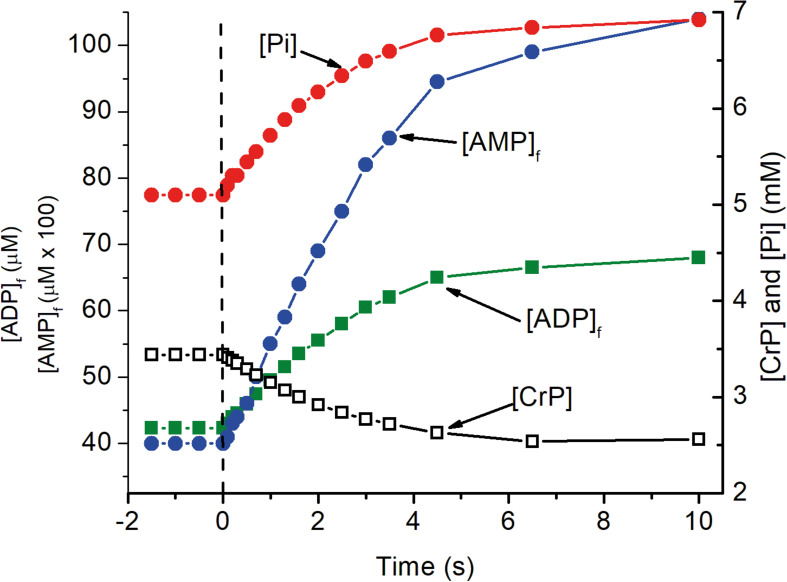
The transition of metabolite levels on as a function of time after activation as simulated in Figure 1. At zero time, indicated by the dashed vertical line, ATP consumption was assumed to increase 5 fold, from 84 to 420 μM ATP s^–1^. The transition in metabolite concentrations indicate half the change, increase or decrease, occurs in about 2 s. As noted for Figure 1, this is dependent on [cyt c] with the half time decreasing in proportional to increase in [cyt c].

The predicted time course for the metabolite changes upon activation are shown in Figure 2. The concentrations of ATP, total creatine (CrP + Cr), and cytochrome c were assumed to be 4.5 mM, 8 mM, and 8 μM, respectively. Activation was assumed to require sustained 5-fold increase in ATP consumption and to occur with no change in local pO_2_. The calculated metabolite concentrations at rest and after activation are consistent with experimental measurements for brain tissue *in vivo*. [ADP]_f_ and [AMP]_f_, as well as the respiratory rate, increase significantly within 0.2 s and are increased by 17, 37, and 90%, respectively, within 1 s. Half maximal changes occur by 2 s. The predicted increase in oxygen consumption and decrease in local pO_2_ are shown in Figure 2. The decrease in local pO_2_ has been predicted assuming: a cytochrome c concentration of 8 μM, a local pO_2_ of 40 μM, a linear relationship between pO_2_ and oxygen consumption, and an initial mean pO_2_ gradient of 2 torr. Increase in oxygen consumption and decrease in pO_2_ begins very quickly after activation (<0.2 s) and transitions to the new steady state in 6–8 s with a half time of about 2 s. Huchzermeyer et al. (2013) reported a 5-fold increase in oxygen consumption when initiating cholinergic gamma oscillations in brain slices from rat hippocampus. It is unlikely that all of the neurons were maximally activated by this treatment, so 5-fold increase is a conservative estimate for activation of individual neurons. Lesser or greater increase would not significantly change the time course, only decrease or increase the changes in metabolite concentrations, oxygen consumption, and local pO_2_. This is consistent with the electrode measurements by Silver (1978).

Nutrients, particularly oxygen, are delivered and waste products removed by blood flowing through the capillaries. Capillaries have mean diameters near 6 microns and the number per mm^2^ of tissue is proportional to the local rate of oxygen/glucose consumption (Pawlik et al., 1981; Sokoloff, 1981; Klein et al., 1986; Lauwers et al., 2008). The average separation in cortex is about 25 microns. As a result, all cells are within 12 microns of at least one capillary and only slightly further from 2 to 3 additional capillaries, all of which contribute to sustaining local pO_2_. To provide for increase in oxygen consumption when small cluster of neurons are activated, flow needs to increase not just in the nearest capillary but in all surrounding capillaries. Flow through terminal arterioles, which supplies blood to the capillaries, is regulated prior to branching into capillaries. As a result, local flow can be only marginally altered by changing resistance in individual capillaries, a proposed role for pericytes (Peppiatt et al., 2006; Hall et al., 2014; Mishra et al., 2016). Increased flow through one capillary in a vascular unit would “steal” flow from other capillaries in the unit because flow through the feeding arteriole would not change. Dynamic regulation of capillary resistance may, however, help to stabilize cellular metabolism by applying controlled modulation of local pO_2_ about the “set point” for metabolic homeostasis. As noted above, the volume of tissue fed by each penetrating arteriole (each vascular unit) is a cylinder approximately 500 μm in diameter and having a volume near 0.17 mm^3^ (Silver, 1978; Blinder et al., 2010; Shih et al., 2015). Cortex has from 40,000 to 120,000 neurons/mm^3^ (Peters, 1987; Tsai et al., 2009), and that means each penetrating arteriole feeds 7,000 to 20,000 neurons. Developing a nutrient delivery system to “feed” individual neurons would be prohibitively complex and have a high energy cost. Evolution has retained the mechanism common to other tissues with primary control at the level of individual penetrating arterioles, rather than developing a more complex system specific to the brain.

## On the Fundamental Requirements for Dynamic Upstream Control of Nutrient Delivery to Cells Within Tissues

**FIGURE 3:**
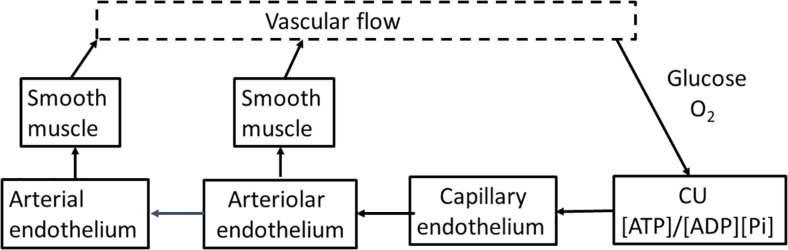
A flow diagram for regulation of cerebrovascular blood flow. Blood flow is regulated through the energy state of functional units made up of metabolically coupled neurons and astrocytes. The energy state provides the set point that is responsible for determining the need for increase or decrease in flow to maintain that set point. The functional units communicate electrically with the capillary endothelium through the astrocyte, altering the endothelial membrane potential. The change in endothelial membrane potential propagates very rapidly (>1 mm/s) along the capillary to the penetrating arteriole and then further into the source arteries. The endothelial cells are coupled with smooth muscle cells that cover the endothelium of the arterial system. As the signal passes along arterial vessels, changes in endothelial membrane potential alter Ca^2+^ levels in the smooth muscle cells. Contraction and relaxation of the smooth muscle controls arteriolar and arterial diameter and thereby blood flow to the tissue.

Basic engineering principles show that effective control of delivery must be applied upstream of where the nutrient is consumed. Flow through arteries and arterioles is determined by vessel diameter, length, applied pressure, and downstream resistance. If input pressure, length, and diameter of the feeding arteriole do not change, only minimal increase in capillary flow can occur. When applied to brain, activation of clusters of neurons within a vascular unit needs to be met by increased flow through the arteries, particularly the penetrating arteriole. We will not attempt to review the literature related to cerebral blood flow but will focus on providing a model/description we believe is consistent with cerebral anatomy, physiology, and biochemistry. The primary requirements for upstream control of blood flow are illustrated in block diagram form in Figure 3. They are: (1) a sensor in tissue that measures deviation from homeostasis in local nutrient concentrations and/or metabolism at the level of individual neurons; (2) a physiological “set point” against which sensor output is compared in order to determine whether upstream regulation should increase or decrease in flow. (3) a mechanism(s) by which changes in sensor output are transmitted upstream from neurons to the arterial system; and (4) a mechanism(s) by which the transmitted signal alters flow through the arterial system that supplies the capillary bed, particularly the terminal arteriole. We will discuss each of parts and then the extent to which available experimental data allows their identification and characterization.

## On the Identity of the Metabolic Sensor That Provides the Measure of Local Metabolism Used to Control Flow

Roy and Sherrington (1890) noted the tight correlation between blood flow and tissue oxygen requirement and concluded that flow is regulated by energy metabolism. Subsequent measurements of cerebral oxygenation and vascular flow have produced data interpreted by some researchers as consistent with, and by others as inconsistent with, regulation by metabolism. Silver (1978), using microelectrodes with a spatial resolution of 3–4 μm (Silver, 1965), reported that in rat brain: “*Oxygen electrodes in the immediate vicinity of inactive neurons showed either a fall, followed by a rise, of pO_2_ when the cell was stimulated, or a rise only. The biphasic pattern was seen only with high resolution electrodes placed close to a cell surface. If a group of electrodes was arranged around the stimulation site, the changes in pO_2_ could not be detected more than 250 μm from the activated cell(s).*” The local decrease in pO_2_ began immediately upon activation (<0.2 s) whereas the increase became evident 1–1.5 s after activation. He also used micro-electrodes to measure several metabolic parameters (pO_2_, pH, K^+^, Cl^–^, Ca^2+^, and lactate) and blood flow in rat brain (Silver, 1978). Local autoregulation and blood flow changes in response to imposed systemic hypoxia were very rapid (1–1.5 s), and increase in blood flow more closely followed pO_2_ than any other parameter. Leniger-Follert and Lübbers (1976) measured pO_2_ and blood flow in response to electrical stimulation and reported both microflow and local oxygen pressure increased after a delay of 1–2 s. No dip in oxygen pressure was observed prior to increase in flow, even when oxygen was measured in a capillary adjacent to the site of electrical stimulation. The authors concluded increase in blood flow could not be due to local increase in metabolism. The spatial resolution of their measurements, however, was approximately 10x lower than those of Silver (1965, 1978). Sokoloff (1981) measured the rate of glucose consumption in different regions of rat brain and concluded the rate of nutrient consumption determined design of the vascular system. Fox and Raichle (1986) using multiple sequential additions of ^15^O-labeled radiotracers and positron emission tomography, measured both cerebral oxygen consumption and cerebral blood flow in humans. In agreement with Silver (1978) and Sokoloff (1981), the resting rate of oxygen consumption was regionally specific and highly correlated with regional blood flow. On the other hand, no transient decrease (dip) in pO_2_, only an increase in blood flow and oxygenation, was observed upon somatosensory stimulation. Based on lack of a transient decrease, Fox and Raichle (1986) hypothesized “*Dynamic, physiological regulation of CBF by a mechanism (neuronal or biochemical) dependent on neuronal firing per se, but independent of the rate of oxygen consumption.”* This observation has been called the BOLD (Blood-Oxygen-Level-Dependent) effect and used (functional MRI) to localize brain regions in which neuronal activity responds to different stimulatory interventions (Fox and Raichle, 1986; Lou et al., 1987; Ogawa et al., 1992; Vanzetta and Grinvald, 1999; Grinvald et al., 2000; Ances et al., 2009; Kim and Ogawa, 2012).

The observation that measurements of pO_2_ in regions of brain activated by somatosensory stimulation do not always show a decrease prior to increased blood flow and pO_2_ has fueled ongoing controversy as to whether increased metabolism is responsible for the observed increase in blood flow (Leniger-Follert and Lübbers, 1976; Silver, 1978; Fox and Raichle, 1986; Lou et al., 1987; Ogawa et al., 1992; Vanzetta and Grinvald, 1999; Grinvald et al., 2000; Ances et al., 2001, 2009; Thompson et al., 2003, 2014; Kim and Ogawa, 2012). Interestingly, Silver and Erecińska (1994), using micro-glucose electrodes, reported “*However, when a microelectrode tip was fortuitously located among a group of cells that were activated simultaneously, small and transient falls in the sugar level were recorded, which usually did not exceed 0.1–0.2 mM. The falls were followed by overshoots of similar magnitude above the baseline value before return to the control level*.” This is consistent with activation inducing increase in glucose consumption, like that for oxygen, is localized to a small volume within the vascular unit. Overall, available data indicate that failure to detect stimulation of metabolism prior to increased blood flow can be attributable to insufficient spatial and/or temporal resolution in the measurements (Silver, 1965; Silver and Erecińska, 1994; Vanzetta and Grinvald, 1999; Ances et al., 2001, 2009; Thompson et al., 2003). Vinogradov and coworkers (Finikova et al., 2008; Lebedev et al., 2008; Sakadžić et al., 2010; Devor et al., 2014; Esipova et al., 2019; Şencan et al., 2020) have designed and synthesized phosphorescent oxygen sensors that can be excited by absorption of two photons. Two photon excitation substantially increases the spatial resolution and depth of measurement attainable using phosphorescence quenching, while retaining the method’s intrinsic accuracy, high temporal resolution, and minimal invasiveness. This technology advance is providing greater insight into oxygen delivery to tissue, including the role of cortical vasculature in brain metabolism and function (Sakadžić et al., 2010; Devor et al., 2011, 2014; Moeini et al., 2018, 2019; Li et al., 2019; Şencan et al., 2020).

### Lessons Derived From Studies of Vascular Regulation in Tissues Other Than Brain

As noted above, identification of the metabolic sensor(s) responsible for regulation of blood flow is particularly difficult in brain. This is due to cellular and structural heterogeneity within the tissue as well as limited access *in vivo* due to the protective bone cover. Fortunately, when adapting regulatory mechanisms to specific tissue function, evolution tends to select for the minimum change required to obtain the necessary result. What is learned about regulation of blood flow in other tissues can be used to help infer how brain vasculature is regulated. In heart, as in brain, flow is precisely regulated and effectively responds to large changes in local metabolic rate. Importantly, cells in heart are quite homogeneous and heart itself can be studied *in vitro*, retaining function after removal from the animal. This allows cardiovascular function to be studied while isolated from input from other tissues, simplifying experimental design and facilitating data interpretation. In isolated perfused rat heart (Neely et al., 1976; Giesen and Kammermaier, 1980; Nuutenen et al., 1982, 1983; Starnes et al., 1985), flow correlates with the rate of oxygen consumption whether the change is due to change in work rate or an uncoupler of oxidative phosphorylation is added to the perfusate. On the other hand, when an inhibitor of oxidative phosphorylation is added to the perfusate, flow increases although metabolic rate decreases and oxygen pressure increases (Nuutenen et al., 1982). In all cases, flow correlated with cardiac energy state ([ATP]/[ADP][Pi]) and not with metabolite (oxygen) concentration or metabolic rate. In heart, as in brain, regulation of flow must be implemented upstream of where nutrients are consumed. Nearly all cells in a cardiac vascular unit are myocytes, however, and change metabolic rate together. This eliminates the metabolism-flow mismatch (BOLD effect) that occurs in brain, where only small fraction of the CUs within a vascular unit may be activated. It can be inferred that in brain, as in heart, energy state of cells within the vascular unit is primarily responsible for regulating blood flow. Zonta et al. (2003) reported that in rat cortical slices dilation of arterioles following neuronal activation required glutamate induced Ca^2+^ changes in astrocytes. Blocking the Ca^2+^ changes suppressed dilation whereas activation of individual astrocytes connected to an arteriole with a patch electrode caused vasodilation. Activation of neurons also involves their associated astrocytes and this is consistent with astrocytes contributing to neuronal coupling to the endothelial cells and thereby to arteriolar blood flow.

Living cells require a continuous supply of metabolic energy, and energy metabolism requires more metabolites and produces more waste products than any other metabolic pathway. Energy (ATP) supply supports all energy dependent reactions essential for cell survival. This include synthesis of macromolecules (proteins, DNA, RNA, lipids, and small molecules not present in sufficient amounts in the environment), molecular and ion transport required for maintenance of intracellular environment, and physical movement. Cellular energy state is a universal indicator of cell viability and it is reasonable to expect that regulation is designed to meet the requirements of energy metabolism. In higher organisms, mitochondrial oxidative phosphorylation provides a particularly stable, tightly regulated, energy state with an intrinsic “set point” for metabolic homeostasis (Wilson, 2017; Wilson and Matchinsky, 2019; Wilson and Matschinsky, 2019). Among the small molecules associated with energy state that provide signals used for maintaining homeostasis are ADP and AMP, particularly the latter. These molecules interact with, and alter, the activity of most of the important enzymes in metabolism and of many regulatory factors responsible for maintaining metabolic homeostasis and cell growth. AMP is a particularly sensitive indicator of energy metabolism. Through the activity of adenylate kinase, [AMP] changes as [ADP]^2^, and evolution has made use of this sensitivity as a core, widely used regulator of metabolism. An important example is AMPK, which is well established as having a central role in regulating energy metabolism as well as many related cellular functions (Mihaylova and Shaw, 2012; Auciello et al., 2014; Hardie et al., 2016; Hardie, 2018; Wilson, 2017; Wilson and Matchinsky, 2019; Wilson and Matschinsky, 2019). Because higher organisms are intrinsically complex, however, and extensively interact with their environment, many ancillary levels of control have been added. These ancillary control mechanisms operate “on top of” the core mechanisms primarily responsible for maintaining metabolic homeostasis. Because the ancillary effectors are numerous and their effect can be experimentally enhanced, such as by use of pharmacological doses of drugs, they often obscure the role of core regulators. In isolated perfused heart, for example, there are a wide range of ancillary regulatory factors that can alter flow and result in ambiguity in experimental design and difficulty in data interpretation. Starnes et al. (1985) noted that in isolated heart perfusate flow can be affected by: “*adenosine, [H^+^], key intermediary metabolites, K^+^, catecholamines, serotonin, histamine, Ca^2+^, prostaglandins, prostacyclins, etc.*” These and more also affect blood flow in other tissues, including brain. Our focus is on what is known and/or can be hypothesized about the signal generated by energy metabolism and how that signal regulates blood flow.

### Contralateral vs Ipsilateral Somatosensory Neural Stimulation

It was early observed that stimulation of somatosensory nerves results in changes in blood flow and oxygenation in both the contralateral and ipsilateral hemispheres, and that these are in opposite directions (Silver, 1965; Devor et al., 2008). This has been often observed but without attribution of much significance. It may, however, be important to how the brain functions. From a computational standpoint, one advantage is readily evident. Brain neural activity is intrinsically noisy. Having neural input act on two different regions and generate signals with opposite signs, one positive and the other negative, improves read fidelity. Connection of the regions with a comparator circuit leads to improved signal to noise and allows exclusion (and/or alternate interpretation of) signals that present with only positive or negative values. An additional advantage is that increased oxygen consumption in one region of the brain is compensated for by decreased consumption in another region. This means substantial local changes in neural activity can be accommodated without large changes in net blood flow, i.e., at nearly constant flow to the brain.

## Observation and Characterization of Upstream Vascular Control

Duling and coworkers (Segal and Duling, 1986; Xia and Duling, 1995; Xia et al., 1995) reported that in hamster cheek pouch local transient depolarization induced by K^+^ or phenylephrine are transmitted for mm distances along arterioles. Intracellular electrode measurements showed smooth muscle and endothelial cells are electrically coupled and depolarization affects both cell types. Dietrich and Tyml (1992) demonstrated that in frog sartorius muscle application of norepinephrine to a locus on a capillary resulted in restriction of flow in the feeding arteriole by a signal transmitted 500–600 μm along the capillary. The signal was insensitive to tetrodotoxin (not neural transmission) but blocked by damage to the capillary endothelium between the application site and arteriole. Dietrich et al. (1996) studied the penetrating arterioles in rat brain using several agents for which local application altered flow through the upstream arteriole. Some differences in the rate of transmission along arterioles was noted, but typical rates were greater than 1,000 μm/s. Emerson and Segal (2000) then reported that endothelial hyperpolarization, and accompanying vasodilation, induced by transient local application of acetylcholine to arterioles was rapidly transmitted along endothelium. Smooth muscle cells were not involved in signal transmission *per se* but were required for modulating vascular resistance. Segal and Jacobs (2001) further observed, in arterioles of hamster skeletal muscle, that neither propagation of the hyperpolarization induced by acetylcholine nor that induced by muscle contraction passed through points where endothelium was damaged. They concluded: “*The present findings thereby illustrate a key role for conduction along the endothelium in promoting muscle blood flow during exercise. This signaling pathway is intrinsic to the resistance vasculature and provides a highly responsive mechanism for coordinating the dilatation of feed arteries with the metabolic demands of skeletal muscle fibers*.” Moreover, signals from capillaries are integrated, i.e., when simultaneously applied to two capillaries with the same feeder arteriole the upstream effect is greater than for application to one capillary (Song and Tyml, 1993). Current data indicate individual neurons/astrocytes interact with more than one capillary and signals from only a small number of capillaries is sufficient for altering flow through a vascular unit. Braunstein et al. (2009) measured uptake of Ca^2+^ in endothelial and smooth muscle cells of rat mesenteric arterioles in response to local KCl application. Depolarization induced an increase in Ca^2+^ that began within about 1 s and peaked about 4 s after KCl application as measured at both local and remote locations along the arteriole. The magnitude of the Ca^2+^ change decreased with increase in distance from the site of KCl application. Inhibition of Ca^2+^ entry by micro-application of channel blocker, nifedipine, or NNC 55-0396, between the local and the remote sites blocked calcium uptake at that point but not signal transmission to the remote site. Inward Ca^2+^ currents through voltage dependent calcium channels were necessary for vasoconstriction but not for transmission of signal along the endothelium, for which K^+^ channels are likely responsible (Welsh et al., 2018; Moshkforoush et al., 2020). The transmission speed is high enough to generate physiologically “immediate” (<0.1 s) responses over distances >500 μm. The observed delay between increase in neural activity and increase in blood flow is consistent with the flow depending on coupling of smooth muscle tension to intracellular Ca^2+^. ***Note: We have discussed only the primary fast signal transmission (*>1 *mm/s) that occurs along the endothelium of arteries and capillaries. Signals with slower transmission have also been observed* (Cai et al., 2018) *and may represent another level of control.***

## On the Primary Physiological Basis for Regulation of Coronary Blood Flow

Blood flow is regulated by changes in diameter of vessels in the arterial tree, particularly that of terminal arterioles. Some aspects of the physiological bases for initiation and transmission of regulatory signal upstream (note: transmission is bidirectional but upstream has primary importance in regulating blood flow) from the site of altered metabolic activity remain uncertain. A schematic for control of vascular flow is shown in Figure 3. Changes in metabolic activity of neurons/astrocytes cause increased or decreased polarization of endothelial cells of the nearest capillary(s). Changes in endothelial polarization are transmitted along capillaries to the source arteriole. Endothelial and smooth muscle cells in arterial walls are electrically coupled, and endothelial polarization determines Ca^2+^ levels in the connected smooth muscle cells. This induces contraction or relaxation as needed to attain appropriate flow through the vascular unit. Activation of a few neurons, and their associated astrocytes, within a vascular unit results in: (1). Increase in local metabolic activity as measured by increase in oxygen consumption, decrease in local pO_2_, and decrease in energy state. The metabolic changes become significant very quickly after neuronal activation (<0.2 s). (2) Increased neuronal (and astrocytic) metabolism and their associated decrease in energy state results in increase in membrane potential of neighboring capillary endothelial cells. (3). Endothelial hyperpolarization propagates rapidly (>1 mm/s) along the capillary. (4). When endothelial polarization changes reach the penetrating arteriole, intracellular Ca^2+^ in smooth muscle cells electrically coupled to the endothelium begins to fall in 1.5–2 s, relaxing the muscle. (5). Arteriolar dilation increases intracapillary pressure, blood volume, and flow throughout the vascular unit. (6). Endothelial cell hyperpolarization continues to spread up the arterial tree but with diminishing amplitude as it moves away from the source. This upstream vasodilation in the arterial tree acts to maintain pressure at the penetrating arteriole in order to support increased flow through the vascular unit. The increase in flow is largely independent of how many neurons are activated in the vascular unit, resulting in flow/metabolism mismatch observed as the BOLD effect. Decreased neuronal activity reverses this process with decrease in metabolic rate, increase in energy state, endothelial depolarization, increased Ca^2+^ uptake in smooth muscle, and arteriolar and arterial constriction.

### Some Remaining Questions Concerning Cerebrovascular Blood Flow and Its Regulation

It has not been established how metabolic changes induced by neuronal/astrocytic activation are coupled to endothelial membrane potential. As noted earlier, coupling does not involve the concentration of metabolites *per se* (glucose, oxygen) but their metabolism. Although there is good evidence myocardial energy state is primarily responsibility for regulating flow in heart (Neely et al., 1976; Giesen and Kammermaier, 1980; Nuutenen et al., 1982, 1983; Starnes et al., 1985), much remains to be learned about how the energy state is coupled to endothelial membrane potential. In brain, endothelial response is coupled to neuronal activity, which involves both neurons and astrocytes. It would be surprising, however, if the coupling mechanisms in heart and brain were not very similar.

### Cerebrovascular Function and Special Vulnerabilities

It is common knowledge that brain is particularly vulnerable to both traumatic injury and age-related pathologies. The role of the cerebrovascular system in that vulnerability has been, and is being, extensively studied.

#### Traumatic Brain Injury

The sensitivity to brain vasculature to traumatic injury is different from that of other tissues. Penetrating arterioles bud from surface arteries and travel at 90^o^ away from the skull through the cortex. These vessels are thin (about 10–20 μm diameter) and the walls consist of only two layers, endothelium coated with single layer of smooth muscle. Sudden movement of the skull caused by an impact displaces the solid structure relative to soft cortical tissue. Inertia of the soft tissue results in its being impacted by the solid bone and the resulting spreading of soft tissue at the impact site places maximal shear stress on the penetrating arterioles. As noted earlier, due to lack of collateral circulation among cortical vascular units, rupture of a penetrating arteriole results in death of the cells within that vascular unit. This causes irreversible loss of a small volume of brain tissue. Although the function(s) lost can be taken over by other parts of the cortex, each blocked penetrating arteriole results in additional loss of cortical tissue and some degradation of function. As tissue loss accumulates, so does the potential for cognitive deficit.

#### Aging Is Accompanied by Progressive Hypoxia, a Substantial Contributor to Age Related Brain Dysfunction

**FIGURE 4:**
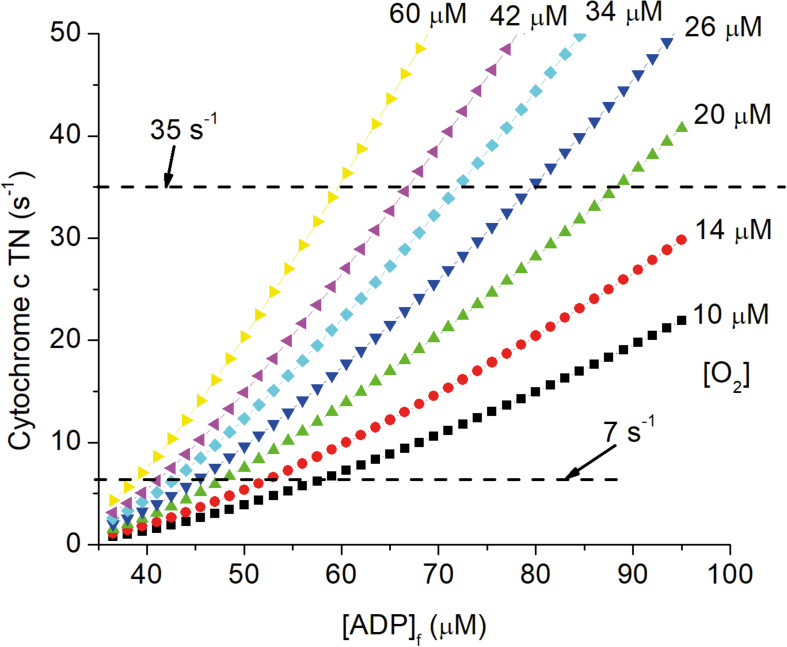
Dependence of cytochrome c turnover (respiratory rate) on [ADP]_f_ and oxygen concentration. The simulation described in the legends of Figures 1, 2, 5 have been carried out for local oxygen concentrations from 10 μM to 60 μM. Cytochrome c TN is plotted against [ADP]_f_ and dashed lines are drawn for cytochrome c TNs of 7 s^–1^ (resting) and 35 s^–1^ (activated) CUs.

**FIGURE 5:**
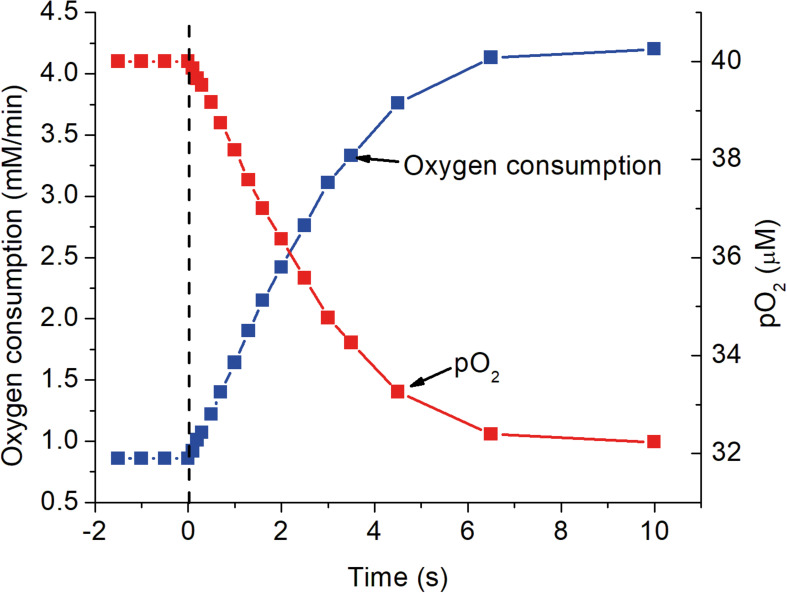
The response of mitochondrial oxygen consumption and local pO_2_ as a function of time after activation as simulated in the previous figures. At zero time, indicated by the dashed vertical line, ATP consumption was assumed to increase 5-fold, from 84 to 420 μM ATP s^–1^. The increase in oxygen consumption closely follows the increase in [ADP]_f_ shown in Figure 2 and has a half time for the transition of about 2 s. Oxygen consumption was calculated for a cytochrome c concentration of 8 μM and the decrease in pO_2_ assumed the mean oxygen gradient (capillary to mitochondria) under resting conditions was 2 μM and proportional to the rate of oxygen consumption.

The central role of vasculature is to deliver oxygen and other nutrients to cells in all tissues and to maintain these at the levels required to sustain metabolic homeostasis. As animals age, vascular function is progressively compromised due to effects of aging as modified by genetic, lifestyle, and disease related factors. Alterations with increasing age include vascular deformities (Hassler, 1967), decreased vascular reactivity (Mooradian and McCuskey, 1992), and perfusion (Ruitenberg et al., 2005; Wolters et al., 2017; Zhang et al., 2010; Zheng et al., 2019), increase in oxygen extraction (Ances et al., 2009; Zhang et al., 2010; Aanerud et al., 2012; Catchlove et al., 2018), decrease in tissue levels of oxygen (Moeini et al., 2018, 2019; Li et al., 2019), and increased hypoxia signaling through AMPK, HIF-1α, TGF-β, etc. (Iadecola, 2010, 2013; Wierenga et al., 2014). Decrease in local pO_2_ requires decrease in energy state (increase in [ADP]f and [AMP]f) to maintain ATP production and oxygen consumption (Wilson et al., 1979, 1988; Wilson, 2017; Wilson et al., 2006; Wilson and Matchinsky, 2019; Wilson and Matschinsky, 2019). This is illustrated in Figure 4, which shows the effect of changing pO_2_ on the relationship of cytochrome c turnover (respiratory rate) to [ADP]_f_. The simulation parameters are the same as for Figures 1, 2, 5 and horizontal dashed lines have been placed for cytochrome c turnover of 7 s^–1^ (resting) and 35 s^–1^ (activated). At all levels of cellular activity decrease in pO_2_ increases the [ADP]_f_ and [AMP]_f_ required to maintain cellular ATP production. For activated neurons and astrocytes, decreased energy state compromises signaling while for resting cells the increased levels, chronically applied, seriously alter metabolic regulation and thereby cell function. Pastuszko and coworkers (Wilson and Pastuszko, 1986; Wilson et al., 1991; Pastuszko et al., 1993; Huang et al., 1994) applied graded levels of hypoxia to the brain of pigs. Decreasing the oxygen fraction in the inspired gas from 21 to 14, 11, and 9%. pO_2_ in the cortical microvasculature (Oxyphor in blood) decreased from 31–35 Torr to 24, 15, and 4 Torr and extracellular dopamine in striatum increased by 80, 200, and 550%, respectively. Hypoxia has also been reported to increase extracellular levels of glutamate and aspartate in hippocampus (Benveniste et al., 1984; Ikonomidou et al., 1989), and glutamate in white matter of fetal sheep (Loeliger et al., 2003) and rats (Rapino et al., 2005). Excessive extracellular concentrations of these neurotransmitters are typically associated with toxicity: dopamine through increased oxygen radical generation (Olano et al., 1995); glutamate through hyperactivation of excitatory glutamate receptors. Na^+^ dependent transport of neurotransmitter amino acids in brain is coupled to the energy state (Wilson and Pastuszko, 1986) and the decrease in energy state associated with even small decrease in oxygen can result in increased extracellular concentrations. It is widely recognized that chronic restriction of oxygen delivery to brain is associated with increased likelihood of cognitive disorders such as Alzheimer’s (AD; Skoog and Gustafson, 2006; Peers et al., 2007; Mecocci et al., 2018). Peers et al. (2007) reported that exposure of cultured astrocytes to chronic hypoxia (6–48 h, 1–2.5% O_2_) potentiated whole-cell voltage-gated Ca^2+^ currents and increased expression of amyloid β peptide (Aβ). Many other risk factors have been individually associated with AD, including hypertension (de la Torre, 2000; Kalback et al., 2004), cholesterol (Sparks et al., 2000), and obesity (Gustafson et al., 2003), and each factor correlates with compromised oxygen delivery. Chronic reduction of oxygen delivery by other mechanisms, including limitation in lung and heart function are also associated with increased cognitive defects. This includes chronic obstructive pulmonary disease (Incalzi et al., 1993), and decreased oxygen in the inspired gas, as experienced by people living at high altitudes or climbing high mountains (Regard et al., 1989; Hornbein, 2008), as well as experimental animals in hypobaric chambers (Titus et al., 2007). Local oxygen concentration directly affects a wide range of cells and cellular properties both *in vivo* and *in vitro* (Morrison et al., 2000; Chen et al., 2007, 2017; Pistollato et al., 2007; Santilli et al., 2010; Xie et al., 2014; Yang et al., 2017). In addition to metabolically stressed cells being more susceptible to failure if further stressed, where proliferation and maturation are required these are often dependent on the normal physiological pO_2_. In adult brain, although neuronal regeneration is minimal, stem cells are present that regenerate specialized cells, such as pericytes, oligodendrocytes, and astrocytes. Stem cell regeneration and differentiation into different types of cells as well as maturation of different cell types, notably oligodendrocytes, have been reported to be dependent on local oxygen concentration (Morrison et al., 2000; Chen et al., 2007, 2017; Santilli et al., 2010; Tilborg et al., 2016; Yang et al., 2017; Janowska et al., 2018; Tsogtbaatar et al., 2020). The total volume and number of cells per unit volume of cortex decreases with age, and the fraction of cells expressing hypoxia inducible factor-1α (HIF-1α) increases (Rapino et al., 2005). As better measurements of pO_2_ *in vivo* have become available, the critical role of pO_2_ is becoming more widely recognized. Unfortunately, many researchers assume changes in pO_2_ within the physiological range do not affect energy metabolism. Alterations in pO_2_ are assumed to affect energy metabolism only when they fall below a “critical corner” [value differs with investigator but typically less than 5 torr (8 μM)]. This is the logical equivalent of studying diabetes and assuming as long as people are not overtly dysfunctional, glucose concentration is not important. In the case of chronic hypoxia, the effects on energy metabolism can be small enough they cause only mild metabolic stress. Because hypoxia induced stress impinges on nearly every aspect of cell and tissue function, the resulting pathology is determined not by the hypoxia *per se*, but by the characteristics and function of individual cells and of any additional imposed stress. In brain, effects of chronic hypoxia include increased leakiness of vessels, abnormal proliferation and differentiation of stem cells, suppressed maturation of critical cells types (oligodendrocytes for myelination), defective myelination, and suppression of critical repair mechanisms. ***We argue that a major cause of age dependent dysfunction in brain are decreased levels of oxygen and glucose***.

#### Progressive Onset of Brain Hypoglycemia, an Underappreciated Result of Aging

Glucose, although known to be essential to brain function, has not been given as much attention as oxygen. This is partly due to the complexity of the process by which glucose gets from the blood into each cell. Unlike oxygen, which readily diffuses through cell membranes, the membranes are impermeable to glucose and crossing each membrane requires glucose “carried across” on a specific transporter protein. Although glucose transport in brain is by facilitated diffusion (not energy linked) there are several transporters, each with different cell specificity, kinetic characteristic, and regulation. This makes both measurements and interpretations difficult. Daniel et al. (1978) measured the uptake and release of ^14^C labeled glucose by the brain in rats from 2–3 weeks to 64 weeks old. Transport rates maximized in young adults (7–9 weeks old) and then influx fell from 0.97 to 0.57 μmole/min/g tissue and efflux from 0.68 to 0.20 μmole/min/g tissue in 64 months old rats. Glucose consumption (influx-efflux; 0.23 μmole/min/g) was not significantly affected by age. Importantly, the efflux/influx ratio fell significantly from 0.70 to 0.35, consistent with the tissue glucose concentration falling from 70% to 35% of the blood concentration (inferred for facilitated diffusion: fully reversible, no energy coupling). Silver and Erecińska (1994) reported micro-glucose electrode measurements in brain of 4–6 week old rats showed interstitial glucose is 2.4 mM when blood glucose is 7.6 mM. Studies using magnetic resonance imaging (Mason et al., 1992; Bernier et al., 2017) have reported glucose consumption rates consistent with Daniel et al. (1978) and estimates of non-vascular glucose concentration consistent with the results of Silver and Erecińska (1994). Decreased tissue levels of glucose, as for oxygen, requires compensatory changes in metabolic signaling in order to maintain energy state and thereby metabolic homeostasis (Wilson et al., 1979, 2006; Wilson, 2017; Wilson and Matchinsky, 2019; Wilson and Matschinsky, 2019).

#### Role of Age Dependent Changes in the Vasculature on Brain Pathology

Alterations in vascular function are largely responsible for age induced chronic hypoxia/hypoglycemia and these exacerbate age dependent weakening of vascular integrity. Overt vascular contributions to cognitive impairment and dementia are prevalent in the elderly and detected in over 85% of cognitively normal individuals 75 years of age or older (Iadecola, 2013; Corriveau et al., 2016). As noted above, chronic hypoxia has a role in increasing the incidence of vascular failure and augmenting the negative consequences. A partial list of diseases with overt vascular lesions includes Alzheimer’s, dementias, stroke, cerebral white matter disease, cerebral small vessel disease (Fisher, 2010; Gorelick et al., 2011; Østergaard et al., 2015; Pinter et al., 2017; Bagi et al., 2018; Moeini et al., 2018, 2019; Rajani et al., 2018; Nation et al., 2019; Sweeney et al., 2019). The vasculature of brain is continuously exposed to, and contributes to, humoral signaling such as inflammation. It is also responsible for the unique permeability barrier between blood and tissue, the blood-brain barrier (BBB). As a result, vessels in brain are more complex than in other tissues. In addition to endothelium, smooth muscle, and pericytes, there are associated astrocytes and oligodendrocytes. These cells are all subject to oxygen/glucose deficiency induced changes in multiple, interacting, signaling pathways, including AMPK, transforming growth factor-β (TGF-β), and HIF. AMPK is the primary regulator for energy metabolism and through the oxygen/glucose dependence of energy metabolism has a significant role in vascular maintenance and signaling (Shirwany and Zou, 2010; Thompson et al., 2014; Chen et al., 2019). TGF-β receptors TβRI and TβRII are found in all areas of the CNS including cortex, hippocampus, striatum, brainstem and cerebellum (Villapol et al., 2013). In cortical gray matter TGF-β receptors are on neurons, astrocytes and microglia and endothelial cells. The TGF-β superfamily and its downstream targets are important in controlling proliferation, differentiation, maturation, and survival of stem cells and precursors in adult brain (Villapol et al., 2013; Wierenga et al., 2014; Nation et al., 2019; Senatorov et al., 2019). Senatorov et al. (2019) reported that, in aging humans and rodents, BBB breakdown begins by middle age and progresses to the end of life span. Hyperactivation of TGF-β signaling by astrocytes was considered necessary and sufficient to cause neural dysfunction and age-related pathology in rodents. Infusion of serum albumin into young rodent brain (mimicking BBB leakiness) induced astrocytic TGF-β signaling and an aged brain phenotype including aberrant electrocorticographic activity, vulnerability to seizures, and cognitive impairment. Nation et al. (2019) reported that BBB leakiness due to damage to brain capillary pericytes develops early in adults that have cognitive dysfunction independent of Aβ and tau biomarker changes and not associated with detectable inflammation or neuronal degeneration. Moeini et al. (2018, 2019) reported: “*In old mice, low pO_2_ points were co-localized in the form of hypoxic micro-pockets with a size reaching up to ∼200 μm in some regions*.” This is consistent with scar tissue from microbleeds, etc. that result in failure of vascular units. The resulting region of dead tissue is invaded by phagocytic cells and is not vascularized, resulting in both near anoxia and decrease in size. **Vascular malfunction/failure is an important determinant of brain pathology through the intimate relationship between vascular function and tissue nutrient supply**.

## Author Summary

Many age and trauma related brain pathologies can be traced to failure of the vasculature that occurs on three general levels: 1. Traumatic injury causes shear stress in the soft tissue of the brain as impacts the ridged bone of the skull, leading to vascular rupture, particularly of penetrating arterioles. Due to lack of protective collateral flow, penetrating arteriole failure results in death of the cells within the vascular unit and loss of brain tissue. 2. Age dependent changes in vasculature lead to progressive increase in vascular resistance, progressive decrease in tissue levels of oxygen and glucose. 3. Hypoxia/hypoglycemia gradually imposed on the brain causes progressive disruption of regulatory mechanisms, particularly related to energy metabolism. These metabolic alterations interfere with stem cell proliferation and differentiation, undermining vascular integrity and suppressing critical repair mechanisms such as oligodendrocyte generation (from stem cells) and maturation. Reduced structural integrity results in local leakiness (extravasation), which can cause local regions of acute hypoxia and microbleeds, while failure of oligodendrocytes to fully mature leads to poor axonal myelination and defective neuronal function.

Understanding and treating age related pathologies, particularly in the brain, requires better knowledge of why and how vasculature changes with age. That knowledge will, hopefully, make possible development of drugs/methods for protecting vascular function, substantially alleviating the negative health effects associated with growing old.

## Author Contributions

DW was the lead author and wrote most of the manuscript with continuous discussion/critique by FM. Both authors have read and approved the final version.

## Conflict of Interest

The authors declare that the research was conducted in the absence of any commercial or financial relationships that could be construed as a potential conflict of interest.
